# Syndromic Antibiograms and Nursing Home Clinicians’ Antibiotic Choices for Urinary Tract Infections

**DOI:** 10.1001/jamanetworkopen.2023.49544

**Published:** 2023-12-27

**Authors:** Lindsay N. Taylor, Brigid M. Wilson, Mriganka Singh, Jessica Irvine, Sally A. Jolles, Corinne Kowal, Taissa A. Bej, Christopher J. Crnich, Robin L. P. Jump

**Affiliations:** 1 University of Wisconsin School of Medicine and Public Health, Madison; 2 William S. Middleton Veterans Affairs Medical Center, Madison; 3 University of Wisconsin Hospital and Clinics, Madison; 4 Geriatric Research Education and Clinical Center (GRECC), Veterans Affairs Northeast Ohio Healthcare System, Cleveland; 5 Division of Infectious Diseases and HIV Medicine in the Department of Medicine, Case Western Reserve University School of Medicine, Cleveland, Ohio; 6 Division of Geriatrics and Palliative Medicine, Department of Medicine, Warren Alpert School of Medicine at Brown University, Providence, Rhode Island; 7 TECH-GRECC, Veterans Affairs Pittsburgh Healthcare System, Pittsburgh, Pennsylvania; 8 Division of Geriatric Medicine, Department of Medicine, School of Medicine, University of Pittsburgh, Pittsburgh, Pennsylvania

## Abstract

**Importance:**

Empirical antibiotic prescribing in nursing homes (NHs) is often suboptimal. The potential for antibiograms to improve empirical antibiotic decision-making in NHs remains poorly understood.

**Objective:**

To determine whether providing NH clinicians with a urinary antibiogram improves empirical antibiotic treatment of urinary tract infections (UTIs).

**Design, Setting, and Participants:**

This was a survey study using clinical vignettes. Participants were recruited via convenience sampling of professional organization listservs of NH clinicians practicing in the US from December 2021 through April 2022. Data were analyzed from July 2022 to June 2023.

**Interventions:**

Respondents were randomized to complete vignettes using a traditional antibiogram (TA), a weighted-incidence syndromic combination antibiogram (WISCA), or no tool. Participants randomized to antibiogram groups were asked to use the antibiogram to empirically prescribe an antibiotic. Participants randomized to the no tool group functioned as controls.

**Main Outcomes and Measures:**

Empirical antibiotic selections were characterized as microbiologically (1) active and (2) optimal according to route of administration and spectrum of activity.

**Results:**

Of 317 responses, 298 (95%) were included in the analysis. Duplicate responses (15 participants), location outside the US (2 participants), and uninterpretable responses (2 participants) were excluded. Most respondents were physicians (217 respondents [73%]) and had over 10 years of NH practice experience (155 respondents [52%]). A mixed-effects logistic model found that use of the TA (odds ratio [OR], 1.41; 95% CI, 1.19-1.68; *P* < .001) and WISCA (OR, 1.54; 95% CI, 1.30-1.84; *P* < .001) were statistically superior to no tool when choosing an active empirical antibiotic. A similarly constructed model found that use of the TA (OR, 1.94; 95% CI, 1.42-2.66; *P* < .001) and WISCA (OR, 1.7; 95% CI, 1.24-2.33; *P* = .003) were statistically superior to no tool when selecting an optimal empirical antibiotic. Although there were differences between tools within specific vignettes, when compared across all vignettes, the TA and WISCA performed similarly for active (OR, 1.09; 95% CI, 0.92-1.30; *P* = .59) and optimal (OR, 0.87; 95% CI, 0.64-1.20; *P* = .69) antibiotics.

**Conclusions and Relevance:**

Providing NH clinicians with a urinary antibiogram was associated with selection of active and optimal antibiotics when empirically treating UTIs under simulated conditions. Although the antibiogram format was not associated with decision-making in aggregate, context-specific effects may have been present, supporting further study of syndromic antibiograms in clinical practice.

## Key Points

**Question:**

Are urinary antibiograms associated with nursing home clinicians’ empirical antibiotic choices for urinary tract infection?

**Findings:**

In this survey study of 317 nursing home clinicians simulating 4 clinical scenarios representing urinary tract infections in nursing home residents, clinicians provided antibiograms were more likely to choose both active and optimal empirical antibiotics.

**Meaning:**

These findings suggest that urinary antibiograms improve empirical antibiotic choice without increased use of overly broad initial therapy and that syndromic antibiograms should be further developed for use as an antibiotic stewardship tool in nursing homes.

## Teaser

This survey study evaluates whether providing nursing home clinicians with a urinary antibiogram improves empirical antibiotic treatment of urinary tract infections.

## Introduction

Up to 85% of antibiotic treatment courses in nursing homes (NHs) are initiated empirically before results of cultures are available.^1,2,3,4^ Up to two-thirds of these empirical choices are inappropriate and may result in harms from not providing active coverage as well as providing too broad of activity.^1,5,6^ Harms of exposure to multiple antibiotic classes or overly broad therapy include increased risk of developing *C difficile* diarrhea^7^ and future infection by antibiotic-resistant bacteria,^8,9^ as well as spread of resistant bacteria to other NH residents.^10,11^ In NHs, over 90% of performed cultures are from urine samples, and urinary tract infections (UTIs) are the most common indication for antibiotic initiation.^12,13,14^ Improving the quality of empirical antibiotic decisions is a critical step toward improving antibiotic safety and effectiveness in NHs.

Cumulative susceptibility reports, commonly called antibiograms, summarize the susceptibility rates of bacteria recovered from cultures collected in a health care facility over a specific time frame. Clinicians can use these tools to guide empirical antibiotic choices, increasing the likelihood that the first prescribed antibiotic will provide adequate activity while awaiting culture and other diagnostic studies.^15^ Syndromic antibiograms, limited to cultures from a specific body site, can provide more targeted empirical antibiotic recommendations that only include antibiotic choices appropriate to the specific syndrome.^16^ The outcomes of antibiograms specific to bacteria isolated from urine on empirical antibiotics selected by NH clinicians has not been well characterized.

Our objective was to assess how urinary antibiograms may be associated with empirical antibiotics selected by NH clinicians treating clinically suspected UTIs. We hypothesized that NH clinicians using a urinary antibiogram would be more likely to select empirical antibiotics that were active against potential urinary pathogens. We further hypothesized that compared with a traditionally formatted urinary antibiogram,^17^ NH clinicians using a weighted-incidence syndromic antibiogram (WISCA)^18^ would be more likely to select antibiotics that were not only active but also optimal in the treatment of NH residents with clinically suspected UTI. To test our hypotheses, we asked NH clinicians to participate in an electronic survey that included 4 clinical vignettes describing NH residents with UTIs. Survey participants were randomized to use a traditional antibiogram (TA), a WISCA, or no antibiogram when selecting empirical antibiotics for each case.

## Methods

### Study Design and Survey Development

We performed a survey study that included 4 clinical vignettes to determine how 2 differently formatted antibiograms were associated with NH clinicians’ decisions for treating UTIs compared with the absence of an antibiogram. The study was approved by the University of Wisconsin Institutional Review Board and deemed to be minimal risk research. Informed consent was obtained through recruitment emails and the survey tool. This study followed the American Association for Public Opinion Research (AAPOR) reporting guideline.

Several clinical vignettes representing common presentations of UTI in the NH setting were developed with the aim of creating realistic cases where multiple antibiotic choices were reasonable.^19,20^ Through an iterative process that included cognitive interviews with NH clinicians who helped to pilot test the survey,^21^ we refined 4 cases for inclusion in the final survey: (1) uncomplicated acute cystitis in an NH resident with a history of *C difficile* infection, (2) pyelonephritis in an NH resident with an antibiotic allergy, (3) an NH resident with a catheter-associated UTI, and (4) uncomplicated cystitis in an NH resident with a recent history of a drug-resistant urinary isolate (eAppendix in Supplement 1). The vignettes were embedded within a 20-item survey that was pretested for clarity and function among a volunteer sample of resident physicians.

**Figure 1.**
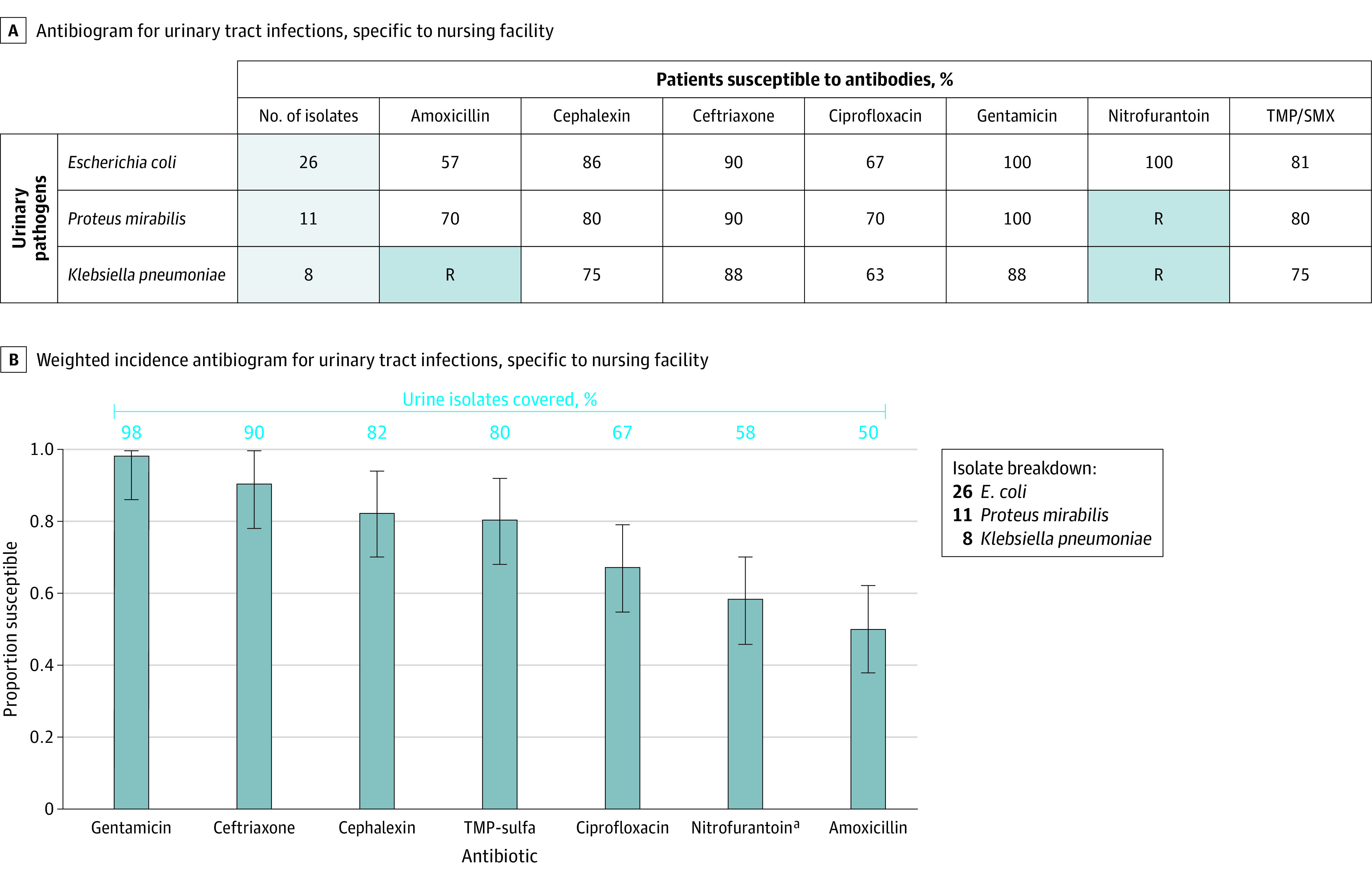
Traditional Antibiogram and Weighted-Incidence Syndromic Antibiogram Used in the Survey The same population of 45 g-negative urinary isolates were used to create both the traditional antibiogram and the weighted-incidence syndromic antibiogram. Light blue shading indicates results based on fewer than 30 isolates, which should be interpreted with caution as they are less reliable. R indicates that the organism is intrinsically resistant. Error bars indicate 95% CI. TMP/SMX indicates trimethoprim/sulfamethoxazole. ^a^Nitrofurantoin had 100% sensitivity for *E coli* but is not active against other listed species.

We used 45 bacterial isolates from 49 urine cultures collected over a 1-year period at a single Wisconsin NH to develop a TA and a WISCA. The formats of both antibiograms underwent iterative revision according to input from content experts and NH clinicians who participated in pilot testing (Figure 1).

### Participant Recruitment and Survey Distribution

Between December 2021 and April 2022, physicians or advanced practice clinicians who prescribe antibiotics in US NHs were recruited via email using a cross-sectional nonprobability convenience sample through professional society listservs and snowball recruiting techniques. Clinicians practicing outside the US, entirely uninterpretable responses, and duplicate responses were excluded. Respondents were reimbursed for their participation ($50 gift card).

The electronically administered survey (Qualtrics) instructed participants to enter an order for a single empirical antibiotic for each case by free text response (eAppendix in Supplement 1). Upon initiating the survey, participants were randomized to use the TA, the WISCA, or no tool when responding to the 4 cases. The TA and WISCA were embedded in the survey such that the case and the antibiogram were viewable on the same screen. All study participants were permitted to use available antibiotic decision-support tools (eg, Sanford Guide) they might use in a clinical encounter. The survey concluded with demographic questions.

### Statistical Analysis

We used descriptive statistics to summarize responses to demographic items. Free-texted antibiotic choices were standardized (ie, misspelled antibiotics or brand names standardized to generic antibiotic names) using nearest neighbor and key collision clustering in Open Refine version 3.5.2 (Google). If more than 1 antibiotic was listed, the first antibiotic was selected as the respondent’s choice. If no antibiotic was listed or if the answer was uninterpretable, it was categorized as missing. Respondents were considered duplicates if they shared identical IP addresses and demographic information. Only the first response from duplicate respondents was included.

**Figure 2.**
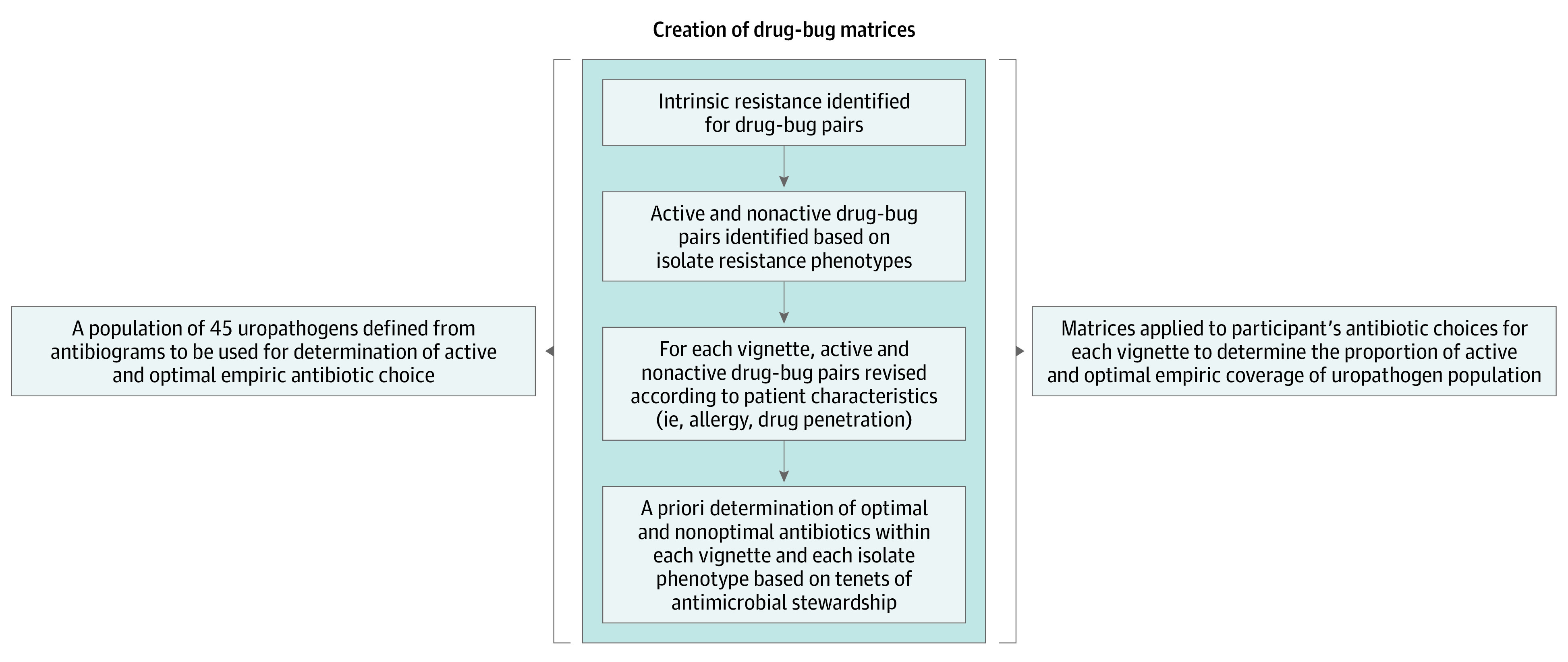
Process Mapping of Methodology Used to Create Bug-Drug Matrices for Active and Optimal Empirical Antibiotic Choice

Assessment of the respondents’ antibiotic choices considered (1) if the selected agent offered active treatment against the population of 45 uropathogens and (2) if the agent represented optimal treatment according to principles of antibiotic stewardship. Active and optimal classifications were operationalized within 4 vignette-specific drug-bug matrices containing all potential combinations of urinary isolates and antibiotic choices for each case (eTable 1 in Supplement 1). These drug-bug matrices were developed from the same population of uropathogens summarized in the TA and WISCA and identified active and optimal antibiotics specific to each isolate resistance phenotype within each vignette according to the process outlined in Figure 2. Active treatment required the antibiotic to be microbiologically active against the pathogen and appropriate for the clinical scenario (eg, antibiotics lacking upper urinary tract penetration were classified as inactive for the vignette describing pyelonephritis; antibiotics to which a patient has a known drug allergy were classified as inactive). Optimal treatment was determined a priori by 3 infectious disease specialists (C.J.C., L.N.T., and R.L.P.J.), who first required the agent to be microbiologically active and then favored narrow spectrum over broad spectrum agents as well as oral antibiotics over those administered parenterally. At least 1 optimal agent was identified for each isolate resistance phenotype. The fourth vignette included a description of a multidrug resistant *Klebsiella* isolate as part of the patient’s recent microbiological history. Accordingly, the bug-drug matrix for this vignette was modified to include the previously recovered isolate and all analyses of this vignette were weighted, assigning 70% weight to the resistant *Klebsiella* isolate and 30% to the other urinary isolates in aggregate.^22,23^ Based on these drug-bug matrices, we calculated the proportion that respondents’ empirical antibiotic choices provided active and optimal coverage for each vignette.

The bug-drug matrices were used to derive 2 binary outcomes for each case’s selected antibiotic against each of the urine isolates summarized in the antibiogram: (1) active or not active and (2) optimal or not optimal. A mixed-effects logistic regression model with selection of an empirical antibiotic active against the organism recovered from urine culture (yes or no) as the outcome, vignette as a covariate, and study group as a fixed effect was created. Each participant was treated as a random effect in this model to account for within-participant association across the vignettes for which they provided an antibiotic choice. A similarly constructed model with selection of an optimal antibiotic agent (yes or no) as the outcome variable was also constructed. In the presence of a detected effect of study group, Tukey-adjusted pairwise comparisons of the 3 groups, averaged across case, were performed to determine which groups differed significantly from each other.

To evaluate for possible differences in study group effects between vignette cases, a second set of mixed-effects logistic regression models estimated differences across the study groups in selecting active or optimal antibiotics while considering the case as a possible effect modifier. This was tested by including an interaction between study groups and cases. Respondents (participants) were considered as a random effect in these models as well. In the presence of a detected interaction effect, Tukey-adjusted pairwise comparisons were performed to summarize study group differences by each case. Finally, respondent demographics were considered for inclusion in the model as fixed effects and compared with models without demographics using likelihood ratio χ^2^ tests. Estimated probabilities for active and optimal antibiotic choices and 95% CIs were calculated as the expit-transformed marginal means and their 95% CIs from the estimated logistic models. All hypothesis tests were 2-sided with significance predefined as an α of .05 or less. All analyses were performed in R Version 4.3.1 (R Project for Statistical Computing) using functions from the lme4 and emmeans packages. Data were analyzed from July 2022 to June 2023.

## Results

### Respondent Characteristics and Observed Responses

Respondents completed 317 surveys, of which 298 (94%) were included in our analysis. The majority of respondents were physicians (217 participants [73%]) and had over 10 years of long-term care practice experience (155 participants [52%]) (Table 1). Selection of an active empirical antibiotic for cases 1 to 3 was higher among respondents randomized to either antibiogram group compared with those in the no tool group (Table 2). For case 4, however, respondents in the WISCA (73 participants) and no tool groups (70 participants) were more likely to select an active antibiotic treatment compared with those in the TA group (46 participants). Selection of an optimal empirical antibiotic for cases 1 to 2 was higher among respondents randomized to use either antibiogram compared with those in the no antibiogram group . Interestingly, respondents using the TA were most likely to select optimal empirical antibiotic therapy for case 3 and least likely to select optimal therapy for case 4. The observed rates of active and optimal therapy differed by vignette, as did the differences between study groups.

**Table 1.** Survey Respondent Characteristics

Characteristic	Participants, No. (%)			
	All respondents (N = 298)^a^	Control (no tool) (n = 101)	Traditional antibiogram (n = 99)	WISCA (n = 98)
Clinician type				
Physician	217 (73)	75 (74)	75 (76)	67 (68)
Advanced practitioner	73 (24)	23 (23)	21 (21)	29 (30)
Not reported	8 (3)	3 (3)	3 (3)	2 (2)
Total practice, y				
<10	88 (29)	28 (28)	22 (22)	38 (39)
≥10	202 (68)	70 (69)	74 (75)	58 (59)
Not reported	8 (3)	3 (3)	3 (3)	2 (2)
Long-term care practice, y				
<10	135 (45)	49 (49)	39 (39)	47 (48)
≥10	155 (52)	49 (49)	57 (58)	49 (50)
Not reported	8 (3)	3 (3)	3 (3)	2 (2)
Specialty				
Geriatrics	125 (42)	43 (43)	42 (42)	40 (41)
Internal medicine	98 (33)	37 (37)	27 (27)	34 (35)
Family medicine	76 (26)	27 (27)	30 (30)	19 (19)
Infectious diseases	1 (1)	0	1 (1)	0
Region				
South	91 (30)	29 (29)	37 (37)	25 (26)
Midwest	86 (29)	26 (26)	27 (27)	33 (34)
Northeast	75 (25)	28 (28)	37 (37)	25 (26)
West	43 (14)	16 (16)	12 (12)	15 (16)
Not reported	3 (1)	2 (2)	1 (1)	0

Abbreviation: WISCA, weighted-incidence syndromic combination antibiogram.

^a^ Nineteen of 317 responses were excluded from analysis due to duplicate response (15 participants), location outside the US (2 participants), and entirely uninterpretable responses (2 participants).

**Table 2.** Observed Active and Optimal Empirical Antibiotic Selections for Each Group and Case

Case	Group, No./total No. (%)		
	No tool	Traditional antibiogram	WISCA
Active therapy			
Case 1	3020/4500 (67.1)	3444/4365 (78.9)	3305/4410 (74.9)
Case 2	2559/4455 (57.4)	2978/4365 (68.2)	2840/4320 (65.7)
Case 3	3008/4410 (68.2)	3180/4410 (72.1)	3410/4320 (78.9)
Case 4	10 466/15 000 (69.8)	6889/14 850 (46.4)	10 463/14 400 (72.7)
Optimal therapy			
Case 1	2435/4500 (54.1)	2679/4365 (61.4)	2914/4410 (66.1)
Case 2	1233/4455 (27.7)	2072/4365 (47.5)	2060/4320 (47.7)
Case 3	1514/4410 (34.3)	2265/4410 (51.4)	1308/4320 (30.3)
Case 4	7258/15 000 (48.4)	3033/14 850 (20.4)	7386/14 400 (51.3)

Abbreviation: WISCA, weighted-incidence syndromic combination antibiogram.

The numbers represent the active and optimal drug-bug pairs for each case. For cases 1 to 3, the denominators represent the product of the number of respondents by the number of isolates with removal of missing responses. For case 4, an additional isolate was included with frequencies equivalent to a 70% weighting vs 30% total weighting of 45 isolates summarized for cases 1 to 3.

### Logistic Models

**Figure 3.**
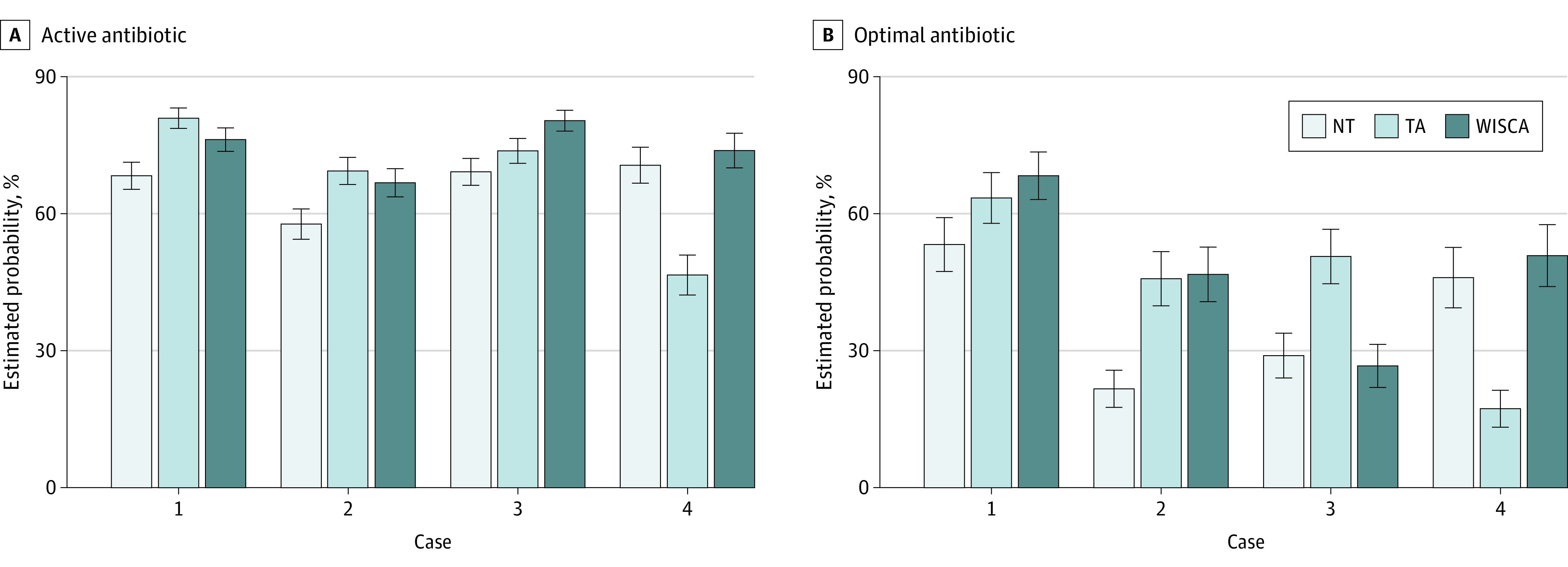
Estimated Mean Probabilities of Active and Optimal Therapy for Each Case by Group Pairwise differences among groups were tested within each outcome and case with Tukey adjustment for the multiple comparisons in each. Error bars indicate 95% CI. NT indicates no tool; TA, traditional antibiogram; WISCA, weighted-incidence syndromic combination antibiogram.

We used mixed-effects logistic models to explore these results in greater detail while adjusting for possibly associated responses within individual participants across the 4 vignettes. We tested a sequence of hypotheses with added model variables (eTable 2 in Supplement 1). We found the treatment groups to be associated with active therapy while adjusting for vignette as a covariate. Specifically, use of either the TA or WISCA was associated with 41% (95% CI, 19%-68%; *P* < .001) and 54% (95% CI, 30%-84%; *P* < .001) greater odds of choosing an active antibiotic compared with no tool, respectively, but the difference between the 2 antibiogram tools was not statistically significant (OR, 1.09; 95% CI, 0.92-1.3; *P* = .59). The addition of demographic variables did not improve the model, but we found that clinical vignette was a significant modifier of the study group effect. Considering the model including the study group-by-vignette interaction and not including demographics as our final model, the differences in the estimated probability of selecting an active empirical antibiotic in each study group and case combination were consistent with the observed responses (Figure 3).

We used the same approach to develop mixed-effects logistic models specific for optimal empirical antibiotic selection and arrived at a similar model (eTable 2 in Supplement 1). We found the treatment group was associated with optimal therapy while adjusting for vignette as a covariate—use of either the TA or WISCA was associated with 94% (95% CI, 42%-166%; *P* < .001) and 70% (95% CI, 24%-133%; *P* = .003) greater odds of choosing an active antibiotic compared with no tool, respectively, but again the difference between the 2 antibiogram tools was not statistically significant (OR, 0.87; 95% CI, 0.64-1.2; *P* = .69). The model-estimated probabilities of optimal therapy were consistent with observed responses while controlling for repeated measures within participants (Figure 3).

## Discussion

The current study shows that, on average, providing NH clinicians with a urine-specific antibiogram, regardless of format, at the time of antibiotic prescribing is associated with an increased likelihood of selecting an antibiotic that is active against pathogens previously recovered in an NH. Use of either antibiogram tool was also associated with increased likelihood of choosing optimal therapy, suggesting these tools not only enhance the likelihood of prescribing an active antibiotic but also minimize the use of broad-spectrum agents which may further reduce adverse effects of antibiotic therapy.

Although the tools did not perform differently from each other overall, the tools performed differently in different clinical scenarios. The most notable example is use of the traditional antibiogram in case 4, which depicts an NH resident with recent prior cystitis from an extended-spectrum β-lactamase–producing *Klebsiella* isolate. In this scenario, choosing an empirical antibiotic informed by the patient’s recent susceptibilities was more likely to result in an active agent.^22,23^ The most commonly chosen antibiotic by clinicians using the TA was ceftriaxone, which was not active against the recently recovered isolate described in the vignette. This may be due to ceftriaxone possessing reasonable activity against the total sample of *Klebsiella* isolates recovered from the NH that was used in the creation of the study TA (Figure 1A), and clinicians randomized to the TA tool may have been less likely to consider the susceptibility of the recently recovered isolate described in the vignette. Recognition of clinical situations for which antibiograms may not improve empirical antibiotic choice is an important finding that merits further evaluation in future studies.

Very little is known about the outcomes of antibiogram use in NHs. As inappropriate prescribing for asymptomatic bacteriuria is a major concern in NHs, interventions to improve antibiotic decision-making have primarily focused on the decision to initiate antibiotics.^24,25,26,27,28^ Studies that do focus on empirical antibiotic choice are primarily focused on avoiding use of select antibiotic agents (eg, fluoroquinolones).^29,30^ Antibiograms have effectively improved antibiotic prescribing in other care settings.^31,32,33,34^ Although other studies have explored feasibility issues around creating antibiograms for NHs,^35,36,37^ the only prior study of the effects of antibiograms in NHs that we are aware of explored the effect of implementing a traditional, nonsyndromic antibiogram in a single NH and was underpowered to determine effect.^1^

We chose to test 2 syndromic antibiogram formats in this study, focusing on empirical prescribing for UTIs instead of traditional, nonsyndromic cumulative susceptibility reports. Urine is the most common source for microbiology cultures and UTI is the leading indication for NH-initiated antibiotics.^38^ Furthermore, previous work suggests that the creation of urinary WISCAs is feasible at the individual NH level.^35^ From a clinical vantage point, summarizing isolates by syndrome better supports empirical antibiotic prescribing, as syndromic antibiograms exclude pathogens from nonurinary sources and antibiotic choices that are not appropriate for UTIs.

### Limitations

Our study has limitations worth discussing. First, we applied the resistance patterns of the population of urinary pathogens summarized in the antibiograms for determination of active and optimal empirical antibiotic therapy. We opted for this approach because our goal was to assess if use of either tool was associated with antibiotic choice. In actual practice, clinicians without a tool may have some sense of local resistance patterns based on experience alone, which may decrease the observed effect of using a tool on active choice. Second, the determination of optimal treatment was based on expert opinion of 3 antimicrobial stewardship and infectious disease experts, which introduces some subjectivity into the outcome of optimal antibiotic choice. Optimal therapy must first be an active antibiotic agent, which links these outcomes. Another analysis approach could focus on differences in broad-spectrum antibiotic therapy as defined by antibiotic classes rather than active and optimal treatment. Furthermore, our methods assume that the case patients’ clinical presentations and urine pathogens do not vary significantly from the patient population and urine pathogens used to develop the antibiogram tools.

We chose to first test these tools in a simulated setting as it allows for a fairly quick evaluation of the antibiograms that can be distributed widely using web-based platforms to busy clinicians. Although we requested respondents to answer as they would practice in real-life, our findings have limited generalizability to real practice. Additionally, our convenience sampling method prevents the determination of a response rate, and it may allow for selection bias, limiting the generalizability of our findings. Although this approach allowed for standardized evaluation of many different clinical scenarios, we focused on 4 common urinary tract syndromes. Additionally, our tools represent a single, fairly susceptible population of urine isolates—it is unclear if using a tool representing a different sample of urine isolates would result in both active and optimal antibiotic choices. Furthermore, we chose to embed the tools within the survey at the point of empirical antibiotic decision-making. In practice, antibiograms are typically available through intranet sites, pocket cards, or print outs and may not be readily used by clinicians in real life because of inaccessibility.^39^ Recent reports describe incorporating antibiograms into the processes of antibiotic prescribing.^40^

## Conclusions

In summary, the current study shows that providing NH clinicians with an antibiogram increases the likelihood of selecting active narrow-spectrum antibiotics when empirically treating clinically suspected UTI. These results justify further study of these tools to support antibiotic stewardship in NHs. Further understanding of which clinical and patient-level factors influence how the tools are used and how they may be optimized to address a wider breadth of clinical situations remains necessary. Guidance to improve empirical antibiotic choice is only 1 component of enhancing antibiotic use. As antibiotic prescribing combines multiple intertwining and often complex decisions, embedding antibiograms within syndrome-specific decision-support tools could combine guidance on empirical antibiotic choice with antibiotic dose and duration.^41^ Other areas to evaluate include how to best develop and implement these tools in NHs, perhaps embedded within antibiotic prescribing decision-support tools.

## Notes

Supplement 1. **eAppendix. **Full Survey Tool **eTable 1. **Matrix of Active and Optimal Therapy **eTable 2. **Description of Mixed-Effects Logistic Models for Active and Optimal Therapy

Supplement 2.
Data Sharing Statement
