# Supplementary material for: Syndromic Antibiograms and Nursing Home Clinicians’ Antibiotic Choices for Urinary Tract Infections
Source: JAMA Netw Open. 2023 Dec 27;6(12):e2349544. doi: 10.1001/jamanetworkopen.2023.49544 (PMC10753399; doi:10.1001/jamanetworkopen.2023.49544)
Supplement: Supplement 1. — eAppendix. Full Survey Tool eTable 1. Matrix of Active and Optimal Therapy eTable 2. Description of Mixed-Effects Logistic Models for Active and Optimal Therapy [file jamanetwopen-e2349544-s001.pdf]

Supplemental Online Content

Taylor LN, Wilson BM, Singh M, et al. Syndromic antibiograms and nursing home clinicians' antibiotic choices for urinary tract infections. *JAMA Netw Open*. 2023;6(12):e2349544. doi:10.1001/jamanetworkopen.2023.49544

eAppendix. Full Survey Tool
eTable 1. Matrix of Active and Optimal Therapy
eTable 2. Description of Mixed-Effects Logistic Models for Active and Optimal Therapy

This supplemental material has been provided by the authors to give readers additional information about their work.

© 2023 Taylor LN et al. *JAMA Network Open.*

eAppendix. Full Survey Tool

AHRQ Vignette Survey

Start of Block: Survey Introduction

Survey Overview
We are conducting a survey to learn more about antibiotic prescribing preferences for urinary tract infections in nursing home residents. This study is being conducted by Christopher Crnich, MD, PhD and Robin Jump, MD, PhD. The survey includes 6 clinical vignettes of nursing home residents with questions about antibiotic initiation and prescribing choice. It should take about 15 minutes to complete.

We recommend taking this survey on a device that is tablet-sized or larger.

Please press the blue arrow on the bottom left to move forward in the survey. You are unable to revisit questions once you have moved past them.

Page Break

© 2023 Taylor LN et al. *JAMA Network Open.*

Informed Consent

Participation in this study is anonymous and voluntary. Participating in this study may not benefit you directly, but it will help us learn more about antibiotic prescribing in the nursing home setting and develop a tool to support empiric antibiotic decisions.

If you participate in the study, you will receive a $50.00 Amazon gift card for your time. The information you will share with us will be kept confidential.

If you have any questions about this study, please contact Lindsay Taylor at ltaylor4@uwhealth.org.

There are qualifications to participate in this study: (1) you must be a clinician, either a NP, PA, MD, or DO, and (2) you must currently treat residents of a nursing home. By checking the "I approve" box below, you are indicating that you meet the qualifications above and that you consent to take part in our study.

☐ I approve (4)

End of Block: Survey Introduction

Start of Block: WISCA Intro Page

Please read in its entirety:

Following this screen there will be four clinical vignettes. Each will ask you to pick an antibiotic. Please approach them like you would for any resident of the facilities in which you work. We want this to replicate your practices as closely as possible.

Each case will be presented alongside a novel weighted-incidence syndromic combination antibiogram (WISCA) for your facility pictured here. The WISCA is an alternative to the standard antibiogram format. Rather than presenting antibiotic susceptibilities by bacterial species, a WISCA combines the susceptibilities across all bacteria recovered from a common source (e.g., urine) to predict the activity of individual antibiotics to treat a specific syndrome (e.g., urinary tract infection [UTI]).

© 2023 Taylor LN et al. *JAMA Network Open.*

This instrument was developed using urine culture data from your facility in 2020. Of 114 urine cultures sent during this time period, 54 had no growth, 17 had polymicrobial growth, 7 had *Enterococcus* spp. and 4 had *Candida albicans*. These were consistent with contaminant or colonization. The remainder grew a potentially pathogenic Gram-negative bacteria as detailed in the WISCA.

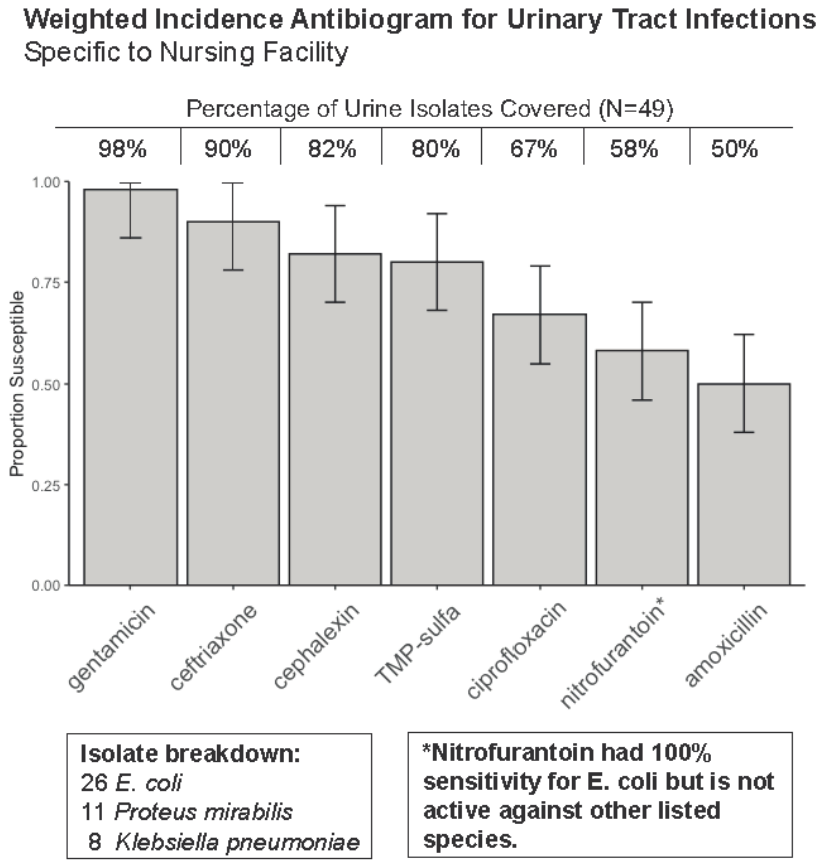

End of Block: WISCA Intro Page

Start of Block: WISCA Abx Choice 1. Cystitis

© 2023 Taylor LN et al. *JAMA Network Open.*

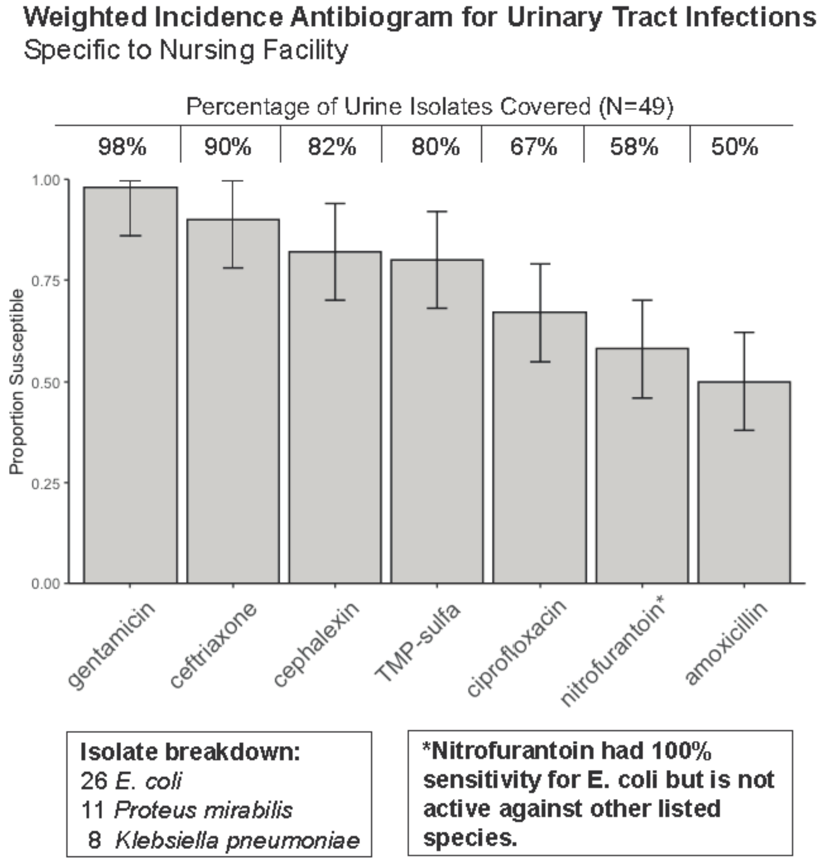

An 87 year-old female with mild dementia, Parkinson's disease on levodopa, diet-controlled diabetes, and stage 3 chronic kidney disease (recent creatine clearance of 50 mL/min) reports new onset dysuria for the last 48 hours. The staff also notes an increase in urinary incontinence. She has no known drug allergies and is able to take pills orally. She had a *Clostridioides difficile* infection about 9 months ago. She has not had any antibiotics in the most recent 3 months.

Her temperature is 98.4°F. Her blood pressure is 118/72 with a heart rate of 64, normal respiratory rate and oxygen saturation of 100% on room air. Her physical exam is notable only for slightly masked facies, mild cogwheel rigidity, a shuffling gait, and benign abdominal exam. Urinalysis shows 3+ pyuria, 2+ nitrites. The urine culture is pending.

© 2023 Taylor LN et al. *JAMA Network Open.*

Assuming you have already decided to treat this patient for UTI, which antibiotic would you prescribe?
Pick a single antibiotic. Dose and length of therapy are not required.

____

Please provide a brief description of your rationale for the antibiotic selection, including reason(s) for the antibiotic selected.

____

____

____

____

____

End of Block: WISCA Abx Choice 1. Cystitis

© 2023 Taylor LN et al. *JAMA Network Open.*

Start of Block: WISCA Abx Choice 2. Pyelonephritis, cephalosporin allergy

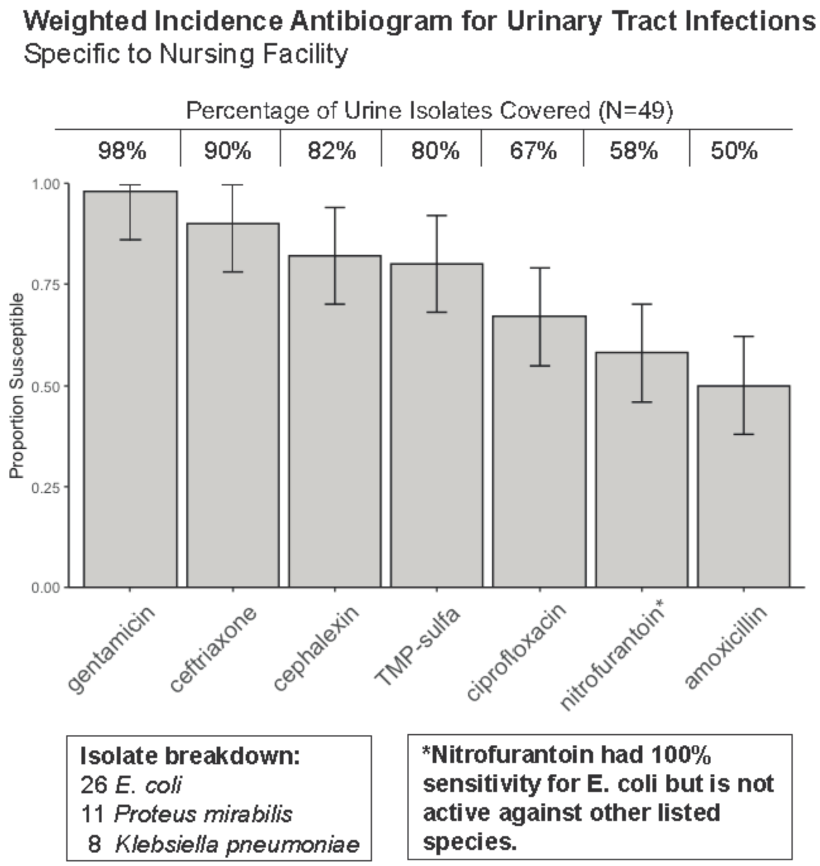

A 78-year-old female with obesity, chronic obstructive pulmonary disease, heart failure, and peripheral vascular disease required an urgent below-knee amputation for limb-threatening ischemia. Her surgery was 2 weeks ago. She has been getting wound care and physical therapy in your building for the previous 10 days. She reports new pain with urination and urgency that developed in the last 48 hours.

Her temperature is 99.2°F. Her blood pressure is 122/82 with a heart rate of 74, normal respiratory rate and oxygen saturation of 100% on room air. Her physical exam is notable for costovertebral angle tenderness to percussion. Based on labs prior to transfer,

© 2023 Taylor LN et al. *JAMA Network Open.*

creatinine clearance was 65 ml/min. Urinalysis shows 3+ pyuria, 2+ nitrites, and white blood cell clumps. Urine culture is pending.

She reports having had swollen lips and difficulty breathing after receiving cephalexin 3 years ago. Beside perioperative antibiotics, she has not taken antibiotics in the prior 3 months.

Assuming you have already decided to treat this patient for UTI, which antibiotic would you prescribe?
Pick a single antibiotic. Dose and length of therapy are not required.

____

Please provide a brief description of your rational for the antibiotic selection, including reason(s) for the antibiotic selected.

____

____

____

____

____

End of Block: WISCA Abx Choice 2. Pyelonephritis, cephalosporin allergy

---

© 2023 Taylor LN et al. *JAMA Network Open.*

Start of Block: WISCA Abx Choice 3. Catheter associated urinary tract infection

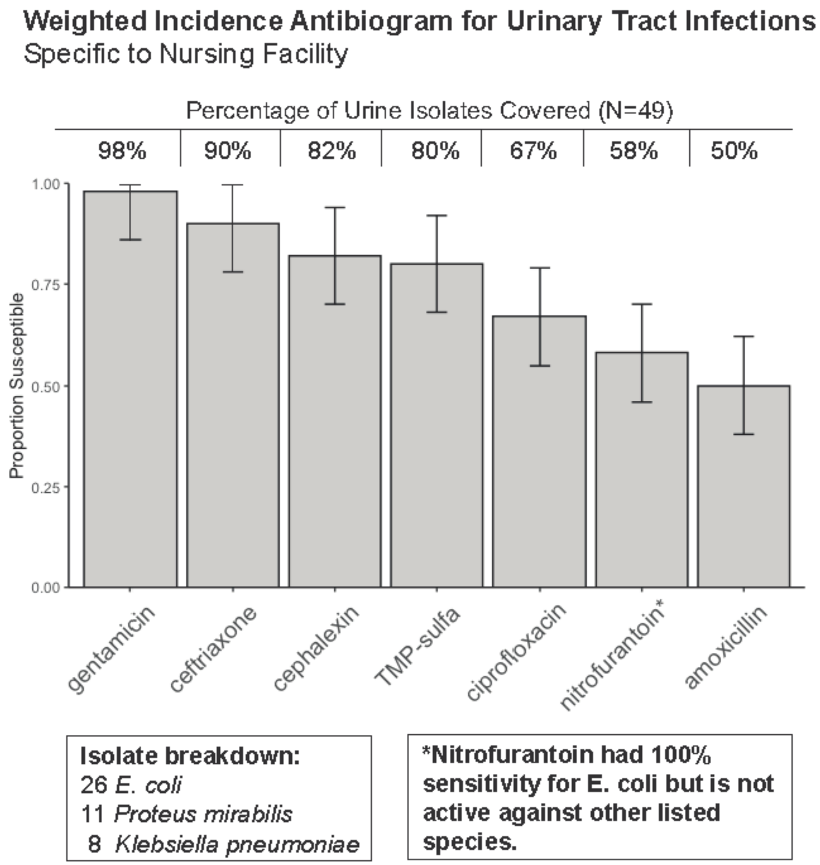

An 83 year-old man with non-insulin dependent diabetes mellitus, hypertension, and benign prostatic hypertrophy with a chronic indwelling urinary catheter has been living in your facility for the last 3 months. This morning, staff noted he was newly confused and did not eat his breakfast but was able to take his morning pills. He has not had any sick contacts and there are no known COVID-19 cases in the facility. The patient has no known antibiotic allergies and has not taken antibiotics in the prior 3 months.

His temperature is 100.4°F. His blood pressure is 135/90 with heart rate of 89, normal respiratory rate and oxygen saturation of 96% on room air. His physical exam is notable for normal cardiac and respiratory exam. He grimaces with applied suprapubic pressure and does not appear to have costovertebral angle tenderness. The chronic indwelling urinary catheter is in place with cloudy urine in

© 2023 Taylor LN et al. *JAMA Network Open.*

the bag. His foley catheter is replaced and urinalysis from a fresh urine sample shows white blood cell clumps, 3+ leukocyte esterase, 3+ nitrites, and many red blood cells. SARS-CoV-2 PCR testing is negative. His most recent creatinine clearance is 65 mL/min.

Assuming you have already decided to treat this patient for UTI, which antibiotic would you prescribe?
Pick a single antibiotic. Dose and length of therapy are not required.

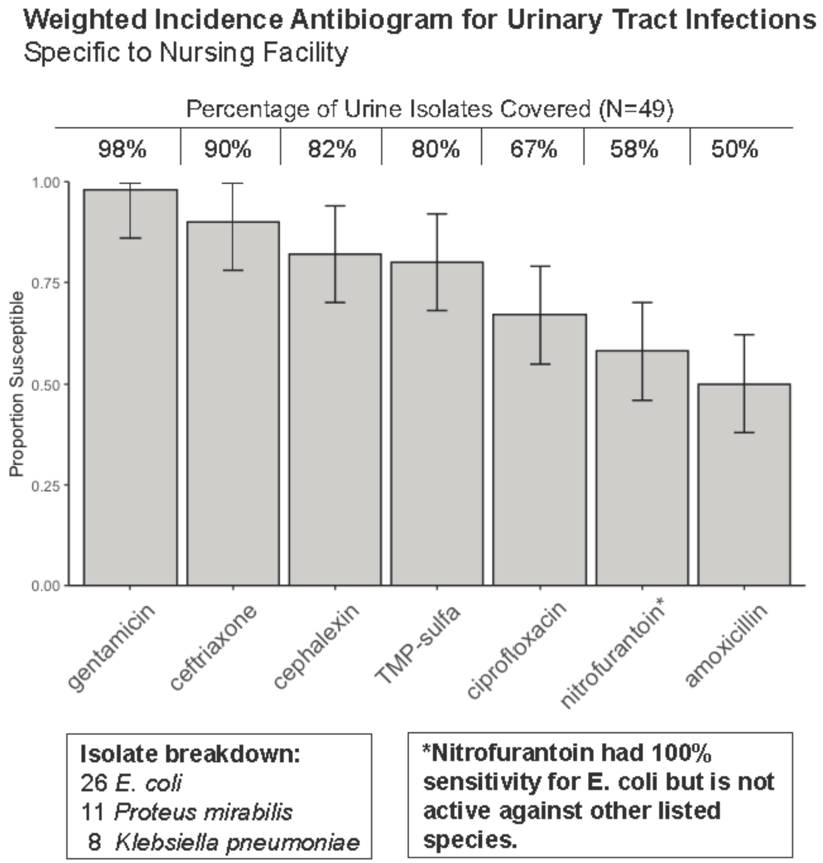

____

© 2023 Taylor LN et al. *JAMA Network Open.*

Please provide a brief description of your rationale for the antibiotic selection, including reason(s) for the antibiotic selected.

____

____

____

____

____

End of Block: WISCA Abx Choice 3. Catheter associated urinary tract infection

---

Start of Block: WISCA Abx Choice 4. Cystitis, history of MDRO

A 73-year-old woman with a history of insulin-dependent diabetes, stroke with associated right sided weakness, and vascular dementia is a resident of your dementia unit. She developed increased urinary urgency, incontinence, and dysuria over the prior 2 days. She does not have a urinary catheter and has no known antibiotic allergies. You recall that 3 months ago she was on contact precautions for an extended-spectrum beta-lactamase (ESBL)-producing Klebsiella species in her urine, and she was treated with ciprofloxacin. Review of that culture shows that the prior Klebsiella isolate was resistant to cefazolin and ceftriaxone, and sensitive to fluoroquinolones, trimethoprim-sulfamethoxazole, nitrofurantoin, cefepime, and carbapenems.

Her temperature is 99.3°F. Her blood pressure is 140/83 with a heart rate of 89, normal respiratory rate and oxygen saturation of 97% on room air. Her physical exam is notable for stable right sided weakness, discomfort with applied suprapubic pressure, and no

© 2023 Taylor LN et al. *JAMA Network Open.*

costovertebral angle tenderness. The urinalysis shows 2+ pyuria and 3+ nitrites. Recent creatinine clearance is 55 mL/min. The urine culture is pending.

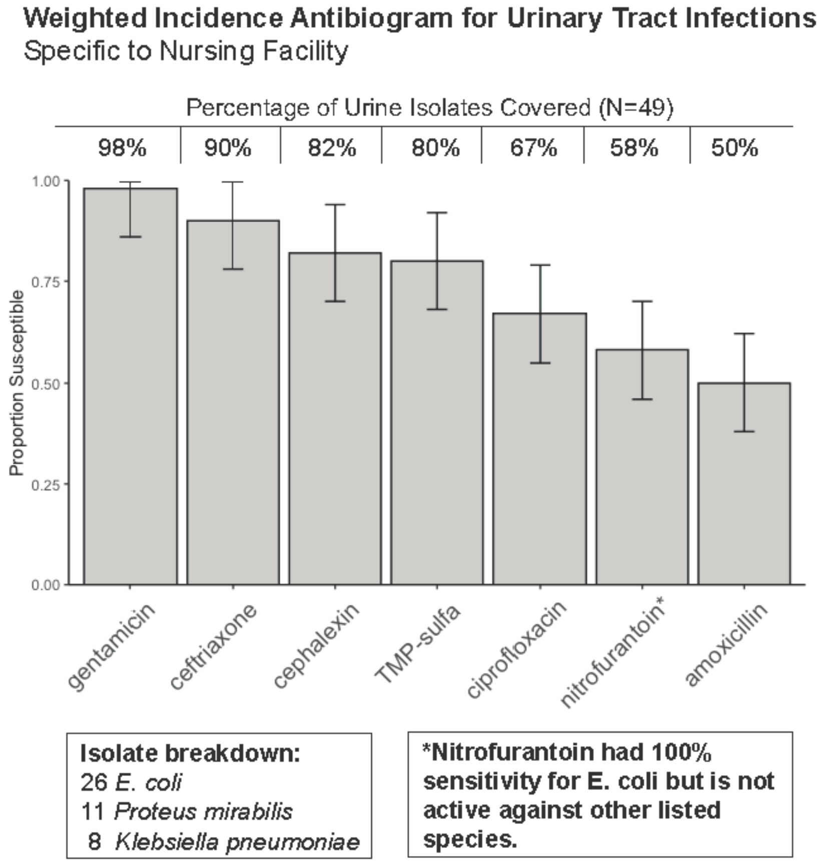

Assuming you have already decided to treat this patient for UTI, which antibiotic would you prescribe?

Pick a single antibiotic. Dose and length of therapy are not required.

____

© 2023 Taylor LN et al. *JAMA Network Open.*

Please provide a brief description of your rationale for the antibiotic selection, including reason(s) for the antibiotic selected.

____

____

____

____

____

End of Block: WISCA Abx Choice 4. Cystitis, history of MDRO

© 2023 Taylor LN et al. *JAMA Network Open.*

Start of Block: Additional Materials Questions

In the prior four cases, which additional materials or tools did you reference while making your antibiotic decisions? (Select all that apply)

- ☐ Online References (4)
- ☐ Local Antibiogram (5)
- ☐ National Guidelines (6)
- ☐ Locally Prepared Guidelines (7)
- ☐ Other (please specify) (8) ____
- ☐ I don't use other reference materials (9)

© 2023 Taylor LN et al. *JAMA Network Open.*

In your practice, which materials or tools do you reference when making UTI treatment decisions for nursing home residents? (Select all that apply)

- ☐ Online References (1)
- ☐ Local Antibiogram (2)
- ☐ National Guidelines (3)
- ☐ Locally Prepared Guidelines (4)
- ☐ Other (please specify) (5) ____
- ☐ I don't use other reference materials (6)

End of Block: Additional Materials Questions

Start of Block: Questions Evaluating Tool Use-WISCA

© 2023 Taylor LN et al. *JAMA Network Open.*

Across the four vignettes, how helpful was the provided weighted-incidence syndromic combination antibiogram in making your antibiotic choice?

- Not At All Helpful (1)
- Slightly Helpful (2)
- Moderately Helpful (3)
- Very Helpful (4)
- Extremely Helpful (5)

Overall, you found interpreting information conveyed in the weighted-incidence syndromic combination antibiogram to be:

- Very Difficult (1)
- Difficult (2)
- Neutral (4)
- Easy (5)
- Very Easy (6)

End of Block: Questions Evaluating Tool Use-WISCA

© 2023 Taylor LN et al. *JAMA Network Open.*

Start of Block: Traditional antibiogram Introduction

Please read in its entirety:
Following this screen there will be four vignettes presented as patient cases. Please approach them like you would for any resident of the facilities in which you work. We want this to replicate your practices as closely as possible.

Each case will be presented alongside the traditional antibiogram pictured here.

This instrument was developed using urine culture data from your facility in 2020. Of 114 urine cultures sent during this time period, 54 had no growth, 17 had polymicrobial growth, 7 had *Enterococcus* spp. and 4 had *Candida albicans*. These were consistent with contaminant or colonization. The remainder grew a potentially pathogenic Gram-negative bacteria as detailed in the antibiogram.

Antibiogram for Urinary Tract Infections
Specific to Nursing Facility

Urinary Pathogens	# of isolates*	Percent Susceptible to Antibiotics (%)						
		Amoxicillin	Cephalexin	Ceftriaxone	Ciprofloxacin	Gentamicin	Nitrofurantoin	TMP/SMX
Escherichia coli	26	57	86	90	67	100	100	81
Proteus mirabilis	11	70	80	90	70	100	R	80
Klebsiella pneumoniae	8	R	75	88	63	88	R	75

*Results based on fewer than 30 isolates are less reliable and should be interpreted with caution.
R denotes that the organism is intrinsically resistant

© 2023 Taylor LN et al. *JAMA Network Open.*

End of Block: Traditional antibiogram Introduction

Start of Block: Traditional Antibiogram Abx Choice 1. Cystitis

An 87 year-old female with mild dementia, Parkinson's disease on levodopa, diet-controlled diabetes, and stage 3 chronic kidney disease (recent creatine clearance of 50 mL/min) reports new onset dysuria for the last 48 hours.

The staff also notes an increase in urinary incontinence. She has no known drug allergies and is able to take pills orally. She had a *Clostridioides difficile* infection about 9 months ago. She has not had any antibiotics in the most recent 3 months.

Her temperature is 98.4°F. Her blood pressure is 118/72 with a heart rate of 64, normal respiratory rate and oxygen saturation of 100% on room air. Her physical exam is notable only for slightly masked facies, mild cogwheel rigidity, a shuffling gait, and benign abdominal exam. Urinalysis shows 3+ pyuria, 2+ nitrites. The urine culture is pending.

Antibiogram for Urinary Tract Infections
Specific to Nursing Facility

Urinary Pathogens	# of isolates*	Percent Susceptible to Antibiotics (%)						
		Amoxicillin	Cephalexin	Ceftriaxone	Ciprofloxacin	Gentamicin	Nitrofurantoin	TMP/SMX
Escherichia coli	26	57	86	90	67	100	100	81
Proteus mirabilis	11	70	80	90	70	100	R	80
Klebsiella pneumoniae	8	R	75	88	63	88	R	75

*Results based on fewer than 30 isolates are less reliable and should be interpreted with caution.
R denotes that the organism is intrinsically resistant

© 2023 Taylor LN et al. *JAMA Network Open.*

Assuming you have already decided to treat this patient for UTI, which antibiotic would you prescribe?
Pick a single antibiotic. Dose and length of therapy are not required.

____

Please provide a brief description of your rationale for the antibiotic selection, including reason(s) for the antibiotic selected.

____

____

____

____

____

End of Block: Traditional Antibiogram Abx Choice 1. Cystitis

Start of Block: Traditional Antibiogram Abx Choice 2. Pyelonephritis, cephalosporin allergy

A 78-year-old female with obesity, chronic obstructive pulmonary disease, heart failure, and peripheral vascular disease required an urgent below-knee amputation for limb-threatening ischemia. Her surgery was 2 weeks ago. She has been getting wound care and physical therapy in your building for the previous 10 days. She reports new pain with urination and urgency that developed in the last 48 hours.

Her temperature is 99.2°F. Her blood pressure is 122/82 with a heart rate of 74, normal respiratory rate and oxygen saturation of

© 2023 Taylor LN et al. *JAMA Network Open.*

100% on room air. Her physical exam is notable for costovertebral angle tenderness to percussion. Based on labs prior to transfer, creatinine clearance was 65 ml/min. Urinalysis shows 3+ pyuria, 2+ nitrites, and white blood cell clumps. Urine culture is pending.

She reports having had swollen lips and difficulty breathing after receiving cephalexin 3 years ago. Beside perioperative antibiotics, she has not taken antibiotics in the prior 3 months.

Antibiogram for Urinary Tract Infections
Specific to Nursing Facility

Urinary Pathogens	# of isolates*	Percent Susceptible to Antibiotics (%)						
		Amoxicillin	Cephalexin	Ceftriaxone	Ciprofloxacin	Gentamicin	Nitrofurantoin	TMP/SMX
Escherichia coli	26	57	86	90	67	100	100	81
Proteus mirabilis	11	70	80	90	70	100	R	80
Klebsiella pneumoniae	8	R	75	88	63	88	R	75

*Results based on fewer than 30 isolates are less reliable and should be interpreted with caution.
R denotes that the organism is intrinsically resistant

Assuming you have already decided to treat this patient for UTI, which antibiotic would you prescribe?
Pick a single antibiotic. Dose and length of therapy are not required.

____

© 2023 Taylor LN et al. *JAMA Network Open.*

Please provide a brief description of your rationale for the antibiotic selection, including reason(s) for the antibiotic selected.

____

____

____

____

____

End of Block: Traditional Antibiogram Abx Choice 2. Pyelonephritis, cephalosporin allergy

---

Start of Block: Traditional Antibiogram Abx Choice 4. Cystitis, history of MDRO

A 73-year-old woman with a history of insulin-dependent diabetes, stroke with associated right sided weakness, and vascular dementia is a resident of your dementia unit. She developed increased urinary urgency, incontinence, and dysuria over the prior 2 days. She does not have a urinary catheter and has no known antibiotic allergies.

You recall that 3 months ago she was on contact precautions for an extended-spectrum beta-lactamase (ESBL)-producing Klebsiella species in her urine, and she was treated with ciprofloxacin. Review of that culture shows that the prior Klebsiella isolate was resistant to cefazolin and ceftriaxone, and sensitive to fluoroquinolones, trimethoprim-sulfamethoxazole, nitrofurantoin, cefepime, and carbapenems.

Her temperature is 99.3°F. Her blood pressure is 140/83 with a heart rate of 89, normal respiratory rate and oxygen saturation of 97% on room air. Her physical exam is notable for stable right sided weakness, discomfort with applied suprapubic pressure, and no

© 2023 Taylor LN et al. *JAMA Network Open.*

costovertebral angle tenderness. The urinalysis shows 2+ pyuria and 3+ nitrites. Recent creatinine clearance is 55 mL/min. The urine culture is pending.

Antibiogram for Urinary Tract Infections
Specific to Nursing Facility

Urinary Pathogens	# of isolates*	Percent Susceptible to Antibiotics (%)						
		Amoxicillin	Cephalexin	Ceftriaxone	Ciprofloxacin	Gentamicin	Nitrofurantoin	TMP/SMX
Escherichia coli	26	57	86	90	67	100	100	81
Proteus mirabilis	11	70	80	90	70	100	R	80
Klebsiella pneumoniae	8	R	75	88	63	88	R	75

*Results based on fewer than 30 isolates are less reliable and should be interpreted with caution.
R denotes that the organism is intrinsically resistant

Assuming that you have already decided to treat this patient, which antibiotic would you prescribe?
Pick a single antibiotic. Dose and length of therapy are not required.

____

Please provide a brief description of your rationale for the antibiotic selection, including reason(s) for the antibiotic selected.

____

© 2023 Taylor LN et al. *JAMA Network Open.*

____

____

____

____

End of Block: Traditional Antibiogram Abx Choice 4. Cystitis, history of MDRO

Start of Block: Traditional Antibiogram Abx Choice 3. Catheter associated urinary tract infection

An 83 year-old man with non-insulin dependent diabetes mellitus, hypertension, and benign prostatic hypertrophy with a chronic indwelling urinary catheter has been living in your facility for the last 3 months. This morning, staff noted he was newly confused and did not eat his breakfast but was able to take his morning pills. He has not had any sick contacts and there are no known COVID-19 cases in the facility. The patient has no known antibiotic allergies and has not taken antibiotics in the prior 3 months.

His temperature is 100.4°F. His blood pressure is 135/90 with heart rate of 89, normal respiratory rate and oxygen saturation of 96% on room air. His physical exam is notable for normal cardiac and respiratory exam. He grimaces with applied suprapubic pressure and does not appear to have costovertebral angle tenderness. The chronic indwelling urinary catheter is in place with cloudy urine in

© 2023 Taylor LN et al. *JAMA Network Open.*

the bag. His foley catheter is replaced and urinalysis from a fresh urine sample shows white blood cell clumps, 3+ leukocyte esterase, 3+ nitrites, and many red blood cells. SARS-CoV-2 PCR testing is negative. His most recent creatinine clearance is 65 mL/min.

Antibiogram for Urinary Tract Infections
Specific to Nursing Facility

Urinary Pathogens	# of isolates*	Percent Susceptible to Antibiotics (%)						
		Amoxicillin	Cephalexin	Ceftriaxone	Ciprofloxacin	Gentamicin	Nitrofurantoin	TMP/SMX
Escherichia coli	26	57	86	90	67	100	100	81
Proteus mirabilis	11	70	80	90	70	100	R	80
Klebsiella pneumoniae	8	R	75	88	63	88	R	75

*Results based on fewer than 30 isolates are less reliable and should be interpreted with caution.
R denotes that the organism is intrinsically resistant

Assuming that you have already decided to treat this patient for UTI, which antibiotic would you prescribe?
Pick a single antibiotic. Dose and length of therapy are not required.

____

Please provide a brief description of your rationale for the antibiotic selection, including reason(s) for the antibiotic selected.

____

© 2023 Taylor LN et al. *JAMA Network Open.*

____

____

____

____

End of Block: Traditional Antibiogram Abx Choice 3. Catheter associated urinary tract infection

---

Start of Block: Questions Evaluating Tool Use- Antibiogram

Across the four vignettes, how helpful was the provided antibiogram in making your antibiotic choice?

- ◯ Not At All Helpful (1)
- ◯ Slightly Helpful (2)
- ◯ Moderately Helpful (3)
- ◯ Very Helpful (4)
- ◯ Extremely Helpful (5)

© 2023 Taylor LN et al. *JAMA Network Open.*

Overall, you found interpreting information conveyed in the antibiogram to be:

- ○ Very Difficult (1)
- ○ Difficult (2)
- ○ Neutral (4)
- ○ Easy (5)
- ○ Very Easy (6)

End of Block: Questions Evaluating Tool Use- Antibiogram

Start of Block: Control Block Introduction

Please read in its entirety:
Following this screen there will be four vignettes presented as patient cases. Please approach them like you would for any resident of the facilities in which you work. We want this to replicate your practices as closely as possible.

End of Block: Control Block Introduction

Start of Block: Control Abx Choice 1. Cystitis

An 87 year-old female with mild dementia, Parkinson's disease on levodopa, diet-controlled diabetes, and stage 3 chronic kidney disease (recent creatine clearance of 50 mL/min) reports new onset dysuria for the last 48 hours. The staff also notes an increase in urinary incontinence. She has no known drug allergies and is able to take pills orally. She had a *Clostridioides difficile* infection about 9 months ago. She has not had any antibiotics in the most recent 3 months.

© 2023 Taylor LN et al. *JAMA Network Open.*

Her temperature is 98.4°F. Her blood pressure is 118/72 with a heart rate of 64, normal respiratory rate and oxygen saturation of 100% on room air. Her physical exam is notable only for slightly masked facies, mild cogwheel rigidity, a shuffling gait, and benign abdominal exam. Urinalysis shows 3+ pyuria, 2+ nitrites. The urine culture is pending.

Assuming you have already decided to treat this patient for UTI, which antibiotic would you prescribe**?**
Pick a single antibiotic. Dose and length of therapy are not required.

____

Please provide a brief description of your rationale for antibiotic selection, including reason(s) for the antibiotic selected.

____

____

____

____

____

End of Block: Control Abx Choice 1. Cystitis

Start of Block: Control Abx Choice 2. Pyelonephritis, cephalosporin allergy

A 78-year-old female with obesity, chronic obstructive pulmonary disease, heart failure, and peripheral vascular disease required an urgent below-knee amputation for limb-threatening ischemia. Her surgery was 2 weeks ago. She has been getting wound care and physical therapy in your building for the previous 10 days. She reports new pain with urination and urgency that developed in the last 48 hours.

Her temperature is 99.2°F. Her blood pressure is 122/82 with a heart rate of 74, normal respiratory rate and oxygen saturation of

© 2023 Taylor LN et al. *JAMA Network Open.*

100% on room air. Her physical exam is notable for costovertebral angle tenderness to percussion. Based on labs prior to transfer, creatinine clearance was 65 ml/min. Urinalysis shows 3+ pyuria, 2+ nitrites, and white blood cell clumps. Urine culture is pending.

She reports having had swollen lips and difficulty breathing after receiving cephalexin 3 years ago. Beside perioperative antibiotics, she has not taken antibiotics in the prior 3 months.

Assuming you have already decided to treat this patient for UTI, which antibiotic would you prescribe?
Pick a single antibiotic. Dose and length of therapy are not required.

____

Please provide a brief description of your rationale for the antibiotic selection, including reason(s) for the antibiotic selected.

____

____

____

____

____

End of Block: Control Abx Choice 2. Pyelonephritis, cephalosporin allergy

---

Start of Block: Control Abx Choice 3. Catheter associated urinary tract infection

An 83 year-old man with non-insulin dependent diabetes mellitus, hypertension, and benign prostatic hypertrophy with a chronic indwelling urinary catheter has been living in your facility for the last 3 months. This morning, staff noted he was newly confused

© 2023 Taylor LN et al. *JAMA Network Open.*

and did not eat his breakfast but was able to take his morning pills. He has not had any sick contacts and there are no known COVID-19 cases in the facility. The patient has no known antibiotic allergies and has not taken antibiotics in the prior 3 months.

His temperature is 100.4°F. His blood pressure is 135/90 with heart rate of 89, normal respiratory rate and oxygen saturation of 96% on room air. His physical exam is notable for normal cardiac and respiratory exam. He grimaces with applied suprapubic pressure and does not appear to have costovertebral angle tenderness. The chronic indwelling urinary catheter is in place with cloudy urine in the bag. His foley catheter is replaced and urinalysis from a fresh urine sample shows white blood cell clumps, 3+ leukocyte esterase, 3+ nitrites, and many red blood cells. SARS-CoV-2 PCR testing is negative. His most recent creatinine clearance is 65 mL/min.

Assuming you have already decided to treat this patient for UTI, which antibiotic would you prescribe?
Pick a single antibiotic. Dose and length of therapy are not required.

____

Please provide a brief description of your rationale for the antibiotic selection, including reason(s) for the antibiotic selected.

____

____

____

____

____

End of Block: Control Abx Choice 3. Catheter associated urinary tract infection

Start of Block: Control Abx Choice 4. Cystitis, history of MDRO

© 2023 Taylor LN et al. *JAMA Network Open.*

A 73-year-old woman with a history of insulin-dependent diabetes, stroke with associated right sided weakness, and vascular dementia is a resident of your dementia unit. She developed increased urinary urgency, incontinence, and dysuria over the prior 2 days. She does not have a urinary catheter and has no known antibiotic allergies. You recall that 3 months ago she was on contact precautions for an extended-spectrum beta-lactamase (ESBL)-producing Klebsiella species in her urine, and she was treated with ciprofloxacin. Review of that culture shows that the prior Klebsiella isolate was resistant to cefazolin and ceftriaxone, and sensitive to fluoroquinolones, trimethoprim-sulfamethoxazole, nitrofurantoin, cefepime, and carbapenems.

Her temperature is 99.3°F. Her blood pressure is 140/83 with a heart rate of 89, normal respiratory rate and oxygen saturation of 97% on room air. Her physical exam is notable for stable right sided weakness, discomfort with applied suprapubic pressure, and no costovertebral angle tenderness. The urinalysis shows 2+ pyuria and 3+ nitrites. Recent creatinine clearance is 55 mL/min. The urine culture is pending.

Assuming you have already decided to treat this patient for UTI, which antibiotic would you prescribe?
Pick a single antibiotic. Dose and length of therapy are not required.

____

Please provide a brief description of your rationale for the antibiotic selection, including reason(s) for the antibiotic selected.

____

____

____

____

____

© 2023 Taylor LN et al. *JAMA Network Open.*

End of Block: Control Abx Choice 4. Cystitis, history of MDRO

Start of Block: Demographic block

What are your credentials?

- ○ Nurse Practitioner (NP) (1)
- ○ Physician Assistant (PA) (2)
- ○ Doctor of Medicine (MD) (3)
- ○ Doctor of Osteopathy (DO) (4)

Display This Question:

If What are your credentials? = Doctor of Medicine (MD)

Or What are your credentials? = Doctor of Osteopathy (DO)

© 2023 Taylor LN et al. *JAMA Network Open.*

What is your specialty and/or sub-specialty training? (Mark all that apply)

- ☐ Family Medicine (1)
- ☐ Internal Medicine (2)
- ☐ Geriatrics (3)
- ☐ Infectious Diseases (4)
- ☐ Other (5)

How long have you been practicing?

- ○ <1 year (1)
- ○ 1-5 years (2)
- ○ 5-10 years (3)
- ○ >10 years (4)

© 2023 Taylor LN et al. *JAMA Network Open.*

How long have you been practicing in long term care?

- <1 year (1)
- 1-5 years (2)
- 5-10 years (3)
- >10 years (4)

End of Block: Demographic block

Start of Block: Conclusion

Thank you for your time and responses!
As noted in the informed consent section, your personal information will not be linked to any survey responses. Please follow the email link below to Corrine Kowal and state your **first and last name** as well as **preferred mailing address** to receive the gift card. The gift card may take 1-2 weeks to arrive.

End of Block: Conclusion

© 2023 Taylor LN et al. *JAMA Network Open.*

eTable 1. Matrix of Active and Optimal Therapy

Matrix of active and optimal therapy for case 1.

Case 1 presents a female nursing home resident with a history of *C. difficile* infection and signs/symptoms consistent with simple cystitis. Isolate-antibiotic combinations are coded in the matrix as follows: inactive antibiotic treatment is indicated by a 0, active but not optimal antibiotic treatment is indicated by a 1, and active and optimal antibiotic treatment is indicated by a 2.

Organism	Susceptibilities	n	amoxicillin	amoxicillin-clavulanate	*cefadroxil	*cefdinir	cefepime	*cefpodoxime	ceftriaxone	cefuroxime	cephalexin	ciprofloxacin	doxycycline	ertapenem	fosfomycin	gentamicin	levofloxacin	macrolide	meropenem	nitrofurantoin	piperacillin-tazobactam	tmp-sulfa
E. coli	GEN=S; CRO=S; CFZ=S; SXT=S; CIP = S; NIT = S; AMX = S; AMC = S; PTZ = S	15	2	1	2	1	1	1	1	1	2	1	0	1	1	1	1	0	1	2	1	2
E. coli	GEN = S; CRO = S; CFZ = S; SXT = S; CIP = S; NIT = S; AMX = R; AMC = S; PTZ = S	2	0	1	2	1	1	1	1	1	2	1	0	1	1	1	1	0	1	2	1	2
E. coli	GEN = S; CRO = S; CFZ = S; SXT = S; CIP = R; NIT = S; AMX = R; AMC = S; PTZ = S	4	0	1	2	1	1	1	1	1	2	0	0	1	1	1	0	0	1	2	1	2
E. coli	GEN = S; CRO = S; CFZ = S; SXT = R; CIP = R; NIT = S; AMX = R; AMC = S; PTZ = S	1	0	1	2	1	1	1	1	1	2	0	0	1	1	1	0	0	1	2	1	0
E. coli	GEN = S; CRO = S; CFZ = R; SXT = R; CIP = R; NIT	1	0	0	0	0	1	0	1	0	0	0	0	1	1	1	0	0	1	2	1	0

© 2023 Taylor LN et al. *JAMA Network Open.*

	= S; AMX = R; AMC = R; PTZ = S																					
E. coli	GEN = S; CRO = R; CFZ = R; SXT = R; CIP = R; NIT = S; AMX = R; AMC = R; PTZ = S	3	0	0	0	0	1	0	0	0	0	0	0	1	1	1	0	0	1	2	1	0
Proteus mirabilis	GEN = S; CRO = S; CFZ = S; SXT = S; CIP = S; NIT = R; AMX = S; AMC = S; PTZ = S	8	2	1	2	1	1	1	1	1	2	1	0	1	1	1	1	0	1	0	1	2
Proteus mirabilis	GEN = S; CRO = S; CFZ = S; SXT = S; CIP = R; NIT = R; AMX = R; AMC = S; PTZ = S	1	0	1	2	1	1	1	1	1	2	0	0	1	1	1	0	0	1	0	1	2
Proteus mirabilis	GEN = S; CRO = S; CFZ = R; SXT = R; CIP = R; NIT = R; AMX = R; AMC = R; PTZ = S	1	0	0	0	0	0	0	2	0	0	0	0	1	1	1	0	0	1	0	1	0
Proteus mirabilis	GEN = S; CRO = R; CFZ = R; SXT = R; CIP = R; NIT = R; AMX = R; AMC = R; PTZ = R	1	0	0	0	0	0	0	0	0	0	0	0	1	2	1	0	0	1	0	0	0
Klebsiella pneumoniae	GEN = S; CRO = S; CFZ = S; SXT = S; CIP = S; NIT = R; AMX = R; AMC = S; PTZ = S	5	0	1	2	1	1	1	1	1	2	1	0	1	1	1	1	0	1	0	1	2

© 2023 Taylor LN et al. *JAMA Network Open.*

Klebsiella pneumoniae	GEN = S; CRO = S; CFZ = S; SXT = S; CIP = R; NIT = R; AMX = R; AMC = R; PTZ = S	1	0	0	2	1	1	1	1	1	2	0	0	1	1	1	0	0	1	0	1	2
Klebsiella pneumoniae	GEN = S; CRO = S; CFZ = R; SXT = R; CIP = R; NIT = R; AMX = R; AMC = R; PTZ = S	1	0	0	0	0	1	0	2	0	0	0	0	1	1	1	0	0	1	0	1	0
Klebsiella pneumoniae	GEN = R; CRO = R; CFZ = R; SXT = R; CIP = R; NIT = R; AMX = R; AMC = R; PTZ = R	1	0	0	0	0	1	0	0	0	0	0	0	1	2	0	0	0	1	0	0	0

Abbreviations: Gentamicin (GEN); Ceftriaxone (CRO); cefazolin (CFZ); trimethoprim-sulfamethoxazole (SXT); ciprofloxacin (CIP); Nitrofurantoin (NIT); Amoxicillin (AMX); amoxicillin-clavulanate (AMC); Piperacillin-tazobactam (TZP)
*Cefazolin is used as a surrogate for cefuroxime, cephalexin, and cefpodoxime.

© 2023 Taylor LN et al. *JAMA Network Open.*

Matrix of active and optimal therapy for case 2.

Case 2 presents a female nursing home resident with history of severe allergy to cephalosporins (angioedema) and signs and symptoms consistent with an upper urinary tract infection (pyelonephritis).

Isolate-antibiotic combinations are coded in the matrix as follows: inactive antibiotic treatment is indicated by a 0, active but not optimal antibiotic treatment is indicated by a 1, and active and optimal antibiotic treatment is indicated by a 2.

Organism	Susceptibilities	n	amoxicillin	amoxicillin-clavulanate	*cefadroxil	*cefdinir	cefepime	*cefpodoxime	ceftriaxone	cefuroxime	cephalexin	Ciprofloxacin	doxycycline	ertapenem	fosfomycin	gentamicin	levofloxacin	macrolide	meropenem	nitrofurantoin	piperacillin-tazobactam	tmp-sulfa
E. coli	GEN=S; CRO=S; CFZ=S; SXT=S; CIP = S; NIT = S; AMX = S; AMC = S; PTZ = S	15	0	0	0	0	0	0	0	0	0	1	0	1	0	1	1	0	1	0	0	2
E. coli	GEN = S; CRO = S; CFZ = S; SXT = S; CIP = S; NIT = S; AMX = R; AMC = S; PTZ = S	2	0	0	0	0	0	0	0	0	0	1	0	1	0	1	1	0	1	0	0	2
E. coli	GEN = S; CRO = S; CFZ = S; SXT = S; CIP = R; NIT = S; AMX = R; AMC = S; PTZ = S	4	0	0	0	0	0	0	0	0	0	0	0	1	0	1	0	0	1	0	0	2
E. coli	GEN = S; CRO = S; CFZ = S; SXT = R; CIP = R; NIT = S; AMX = R; AMC = S; PTZ = S	1	0	0	0	0	0	0	0	0	0	0	0	2	0	2	0	0	1	0	0	0
E. coli	GEN = S; CRO = S; CFZ = R; SXT = R; CIP = R; NIT = S; AMX = R;	1	0	0	0	0	0	0	0	0	0	0	0	2	0	2	0	0	1	0	0	0

© 2023 Taylor LN et al. *JAMA Network Open.*

	AMC = R; PTZ = S																					
E. coli	GEN = S; CRO = R; CFZ = R; SXT = R; CIP = R; NIT = S; AMX = R; AMC = R; PTZ = S	3	0	0	0	0	0	0	0	0	0	0	0	2	0	2	0	0	1	0	0	0
Proteus mirabilis	GEN = S; CRO = S; CFZ = S; SXT = S; CIP = S; NIT = R; AMX = S; AMC = S; PTZ = S	8	0	0	0	0	0	0	0	0	0	1	0	1	0	1	1	0	1	0	0	2
Proteus mirabilis	GEN = S; CRO = S; CFZ = S; SXT = S; CIP = R; NIT = R; AMX = R; AMC = S; PTZ = S	1	0	0	0	0	0	0	0	0	0	0	0	1	0	1	0	0	1	0	0	2
Proteus mirabilis	GEN = S; CRO = S; CFZ = R; SXT = R; CIP = R; NIT = R; AMX = R; AMC = R; PTZ = S	1	0	0	0	0	0	0	0	0	0	0	0	2	0	2	0	0	1	0	0	0
Proteus mirabilis	GEN = S; CRO = R; CFZ = R; SXT = R; CIP = R; NIT = R; AMX = R; AMC = R; PTZ = R	1	0	0	0	0	0	0	0	0	0	0	0	2	0	2	0	0	1	0	0	0
Klebsiella pneumoniae	GEN = S; CRO = S; CFZ = S; SXT = S; CIP = S; NIT = R; AMX = R; AMC = S; PTZ = S	5	0	0	0	0	0	0	0	0	0	1	0	1	0	1	1	0	1	0	0	2

© 2023 Taylor LN et al. *JAMA Network Open.*

Klebsiella pneumoniae	GEN = S; CRO = S; CFZ = S; SXT = S; CIP = R; NIT = R; AMX = R; AMC = R; PTZ = S	1	0	0	0	0	0	0	0	0	0	0	0	1	0	1	0	0	1	0	0	2
Klebsiella pneumoniae	GEN = S; CRO = S; CFZ = R; SXT = R; CIP = R; NIT = R; AMX = R; AMC = R; PTZ = S	1	0	0	0	0	0	0	0	0	0	0	0	2	0	2	0	0	1	0	0	0
Klebsiella pneumoniae	GEN = R; CRO = R; CFZ = R; SXT = R; CIP = R; NIT = R; AMX = R; AMC = R; PTZ = R	1	0	0	0	0	0	0	0	0	0	0	0	2	0	0	0	0	1	0	0	0

Abbreviations: Gentamicin (GEN); Ceftriaxone (CRO); cefazolin (CFZ); trimethoprim-sulfamethoxazole (SXT); ciprofloxacin (CIP); Nitrofurantoin (NIT); Amoxicillin (AMX); amoxicillin-clavulanate (AMC); Piperacillin-tazobactam (TZP)

*Cefazolin is used as a surrogate for cefuroxime, cephalexin, and cefpodoxime.

© 2023 Taylor LN et al. *JAMA Network Open.*

Matrix of active and optimal therapy for case 3.

Case 3 presents a male nursing home resident with a chronic indwelling urinary catheter and signs and symptoms consistent with a catheter-associated urinary tract infection. Additionally, he has fever, raising concern for possible upper urinary tract involvement.
Isolate-antibiotic combinations are coded in the matrix as follows: inactive antibiotic treatment is indicated by a 0, active but not optimal antibiotic treatment is indicated by a 1, and active and optimal antibiotic treatment is indicated by a 2.

Organism	Susceptibilities	n	amoxicillin	amoxicillin-clavulanate	*cefadroxil	*cefdinir	cefepime	*cefpodoxime	ceftriaxone	cefuroxime	cephalexin	Ciprofloxacin	doxycycline	ertapenem	fosfomycin	gentamicin	levofloxacin	macrolide	meropenem	nitrofurantoin	piperacillin-tazobactam	tmp-sulfa
E. coli	GEN=S; CRO=S; CFZ=S; SXT=S; CIP = S; NIT = S; AMX = S; AMC = S; PTZ = S	15	2	1	2	1	1	1	1	1	2	1	0	1	0	1	1	0	1	0	1	2
E. coli	GEN = S; CRO = S; CFZ = S; SXT = S; CIP = S; NIT = S; AMX = R; AMC = S; PTZ = S	2	0	1	2	1	1	1	1	1	2	1	0	1	0	1	1	0	1	0	1	2
E. coli	GEN = S; CRO = S; CFZ = S; SXT = S; CIP = R; NIT = S; AMX = R; AMC = S; PTZ = S	4	0	1	2	1	1	1	1	1	2	0	0	1	0	1	0	0	1	0	1	2
E. coli	GEN = S; CRO = S; CFZ = S; SXT = R; CIP = R; NIT = S; AMX = R; AMC = S; PTZ = S	1	0	1	2	1	1	1	1	1	2	0	0	1	0	1	0	0	1	0	1	0
E. coli	GEN = S; CRO = S; CFZ = R; SXT = R; CIP = R; NIT = S; AMX = R;	1	0	0	0	0	1	0	2	0	0	0	0	1	0	1	0	0	1	0	1	0

© 2023 Taylor LN et al. *JAMA Network Open.*

	AMC = R; PTZ = S																					
E. coli	GEN = S; CRO = R; CFZ = R; SXT = R; CIP = R; NIT = S; AMX = R; AMC = R; PTZ = S	3	0	0	0	0	1	0	0	0	0	0	0	2	0	1	0	0	1	0	1	0
Proteus mirabilis	GEN = S; CRO = S; CFZ = S; SXT = S; CIP = S; NIT = R; AMX = S; AMC = S; PTZ = S	8	2	1	2	1	1	1	1	1	2	1	0	1	0	1	1	0	1	0	1	2
Proteus mirabilis	GEN = S; CRO = S; CFZ = S; SXT = S; CIP = R; NIT = R; AMX = R; AMC = S; PTZ = S	1	0	1	2	1	1	1	1	1	2	0	0	1	0	1	0	0	1	0	1	2
Proteus mirabilis	GEN = S; CRO = S; CFZ = R; SXT = R; CIP = R; NIT = R; AMX = R; AMC = R; PTZ = S	1	0	0	0	0	0	0	2	0	0	0	0	1	0	1	0	0	1	0	1	0
Proteus mirabilis	GEN = S; CRO = R; CFZ = R; SXT = R; CIP = R; NIT = R; AMX = R; AMC = R; PTZ = R	1	0	0	0	0	0	0	0	0	0	0	0	2	0	2	0	0	1	0	0	0
Klebsiella pneumoniae	GEN = S; CRO = S; CFZ = S; SXT = S; CIP = S; NIT = R; AMX = R; AMC = S; PTZ = S	5	0	1	2	1	1	1	1	1	2	1	0	1	0	1	1	0	1	0	1	2

© 2023 Taylor LN et al. *JAMA Network Open.*

Klebsiella pneumoniae	GEN = S; CRO = S; CFZ = S; SXT = S; CIP = R; NIT = R; AMX = R; AMC = R; PTZ = S	1	0	0	2	1	1	1	1	1	2	0	0	1	0	1	0	0	1	0	1	2
Klebsiella pneumoniae	GEN = S; CRO = S; CFZ = R; SXT = R; CIP = R; NIT = R; AMX = R; AMC = R; PTZ = S	1	0	0	0	0	1	0	2	0	0	0	0	1	0	1	0	0	1	0	1	0
Klebsiella pneumoniae	GEN = R; CRO = R; CFZ = R; SXT = R; CIP = R; NIT = R; AMX = R; AMC = R; PTZ = R	1	0	0	0	0	1	0	0	0	0	0	0	2	0	0	0	0	1	0	0	0

Abbreviations: Gentamicin (GEN); Ceftriaxone (CRO); cefazolin (CFZ); trimethoprim-sulfamethoxazole (SXT); ciprofloxacin (CIP); Nitrofurantoin (NIT); Amoxicillin (AMX); amoxicillin-clavulanate (AMC); Piperacillin-tazobactam (TZP)

*Cefazolin is used as a surrogate for cefuroxime, cephalexin, and cefpodoxime.

© 2023 Taylor LN et al. *JAMA Network Open.*

Matrix of active and optimal therapy for case 4.

Case 4 presents a male nursing home resident with a recent history of simple cystitis within the prior 3 months. Urine culture then revealed an extended-spectrum beta-lactamase producing *Klebsiella pneumonia* isolate and treated successfully with ciprofloxacin.

Isolate-antibiotic combinations are coded in the matrix as follows: inactive antibiotic treatment is indicated by a 0, active but not optimal antibiotic treatment is indicated by a 1, and active and optimal antibiotic treatment is indicated by a 2.

Organism	Susceptibilities	n	amoxicillin	amoxicillin-clavulanate	*cefadroxil	*cefdinir	cefepime	*cefpodoxime	ceftriaxone	cefuroxime	cephalexin	Ciprofloxacin	doxycycline	ertapenem	fosfomycin	gentamicin	levofloxacin	macrolide	meropenem	nitrofurantoin	piperacillin-tazobactam	Tmp-sulfa
E. coli	GEN=S; CRO=S; CFZ=S; SXT=S; CIP = S; NIT = S; AMX = S; AMC = S; PTZ = S	15	2	1	2	1	1	1	1	1	2	1	0	1	1	1	1	0	1	2	1	2
E. coli	GEN = S; CRO = S; CFZ = S; SXT = S; CIP = S; NIT = S; AMX = R; AMC = S; PTZ = S	2	0	1	2	1	1	1	1	1	2	1	0	1	1	1	1	0	1	2	1	2
E. coli	GEN = S; CRO = S; CFZ = S; SXT = S; CIP = R; NIT = S; AMX = R; AMC = S; PTZ = S	4	0	1	2	1	1	1	1	1	2	0	0	1	1	1	0	0	1	2	1	2
E. coli	GEN = S; CRO = S; CFZ = S; SXT = R; CIP = R; NIT = S; AMX = R;	1	0	1	2	1	1	1	1	1	2	0	0	1	1	1	0	0	1	2	1	0

© 2023 Taylor LN et al. *JAMA Network Open.*

	AMC = S; PTZ = S																					
E. coli	GEN = S; CRO = S; CFZ = R; SXT = R; CIP = R; NIT = S; AMX = R; AMC = R; PTZ = S	1	0	0	0	0	1	0	1	0	0	0	0	1	1	1	0	0	1	2	1	0
E. coli	GEN = S; CRO = R; CFZ = R; SXT = R; CIP = R; NIT = S; AMX = R; AMC = R; PTZ = S	3	0	0	0	0	1	0	0	0	0	0	0	1	1	1	0	0	1	2	1	0
Proteus mirabilis	GEN = S; CRO = S; CFZ = S; SXT = S; CIP = S; NIT = R; AMX = S; AMC = S; PTZ = S	8	2	1	2	1	1	1	1	1	2	1	0	1	1	1	1	0	1	0	1	2
Proteus mirabilis	GEN = S; CRO = S; CFZ = S; SXT = S; CIP = R; NIT = R; AMX = R; AMC = S; PTZ = S	1	0	1	2	1	1	1	1	1	2	0	0	1	1	1	0	0	1	0	1	2
Proteus mirabilis	GEN = S; CRO = S; CFZ = R; SXT = R; CIP = R; NIT = R; AMX = R; AMC = R; PTZ = S	1	0	0	0	0	0	0	2	0	0	0	0	1	1	1	0	0	1	0	1	0
Proteus mirabilis	GEN = S; CRO = R; CFZ = R; SXT = R; CIP =	1	0	0	0	0	0	0	0	0	0	0	0	1	2	1	0	0	1	0	0	0

© 2023 Taylor LN et al. *JAMA Network Open.*

	R; NIT = R; AMX = R; AMC = R; PTZ = R																					
Klebsiella pneumoniae	GEN = S; CRO = S; CFZ = S; SXT = S; CIP = S; NIT = R; AMX = R; AMC = S; PTZ = S	5	0	1	2	1	1	1	1	1	2	1	0	1	1	1	1	0	1	0	1	2
Klebsiella pneumoniae	GEN = S; CRO = S; CFZ = S; SXT = S; CIP = R; NIT = R; AMX = R; AMC = R; PTZ = S	1	0	0	2	1	1	1	1	1	2	0	0	1	1	1	0	0	1	0	1	2
Klebsiella pneumoniae	GEN = S; CRO = S; CFZ = R; SXT = R; CIP = R; NIT = R; AMX = R; AMC = R; PTZ = S	1	0	0	0	0	1	0	2	0	0	0	0	1	1	1	0	0	1	0	1	0
Klebsiella pneumoniae	GEN = R; CRO = R; CFZ = R; SXT = R; CIP = R; NIT = R; AMX = R; AMC = R; PTZ = R	1	0	0	0	0	1	0	0	0	0	0	0	1	2	0	0	0	1	0	0	0
Klebsiella pneumoniae**	GEN = S; CRO = R; CFZ = R; SXT = S; CIP = S; NIT = S; AMX = R	1	0	0	0	0	1	0	0	0	0	1	0	1	2	1	1	0	1	0	1	2

Abbreviations: Gentamicin (GEN); Ceftriaxone (CRO); cefazolin (CFZ); trimethoprim-sulfamethoxazole (SXT); ciprofloxacin (CIP); Nitrofurantoin (NIT); Amoxicillin (AMX); amoxicillin-clavulanate (AMC); Piperacillin-tazobactam (TZP)

© 2023 Taylor LN et al. *JAMA Network Open.*

*Cefazolin is used as a surrogate for cefuroxime, cephalexin, and cefpodoxime.
**This patient specific isolate presented in this vignette was included in the matrix and weighted more heavily in analysis (70%) to represent the increased likelihood of identifying the same isolate.

eTable 2. Description of Mixed-Effects Logistic Models for Active and Optimal Therapy

Model-tested hypothesis	Outcome = Active Therapy	Outcome = Optimal Therapy
There is a difference in outcome between study arms, adjusting for case as covariate and correlated responses within subject.	Active therapy associated with study arm and case ($p < 0.001$)	Optimal therapy associated with study arm and case ($p < 0.001$)
The antibiograms differ from no antibiogram and from each other.	TA and WISCA higher vs. no antibiogram ($p < 0.001$) but not different from each other	TA and WISCA higher vs. no antibiogram ($p < 0.01$) but not different from each other
The differences in outcomes between arms is not the same for all 4 cases.	Significant case-by-arm interaction effect detected ($p < 0.001$)	Significant case-by-arm interaction effect detected ($p < 0.001$)
Adjusting for demographic factors improves the model fit.	No significant improvement in model fit with addition of demographics (Likelihood ratio $p = 0.91$)	No significant improvement in model fit with addition of demographics (Likelihood ratio $p = 0.12$)

© 2023 Taylor LN et al. *JAMA Network Open.*
