# Supplementary material for: Syndromic Antibiograms and Nursing Home Clinicians’ Antibiotic Choices for Urinary Tract Infections
Source: JAMA Netw Open. 2023 Dec 27;6(12):e2349544. doi: 10.1001/jamanetworkopen.2023.49544 (PMC10753399; doi:10.1001/jamanetworkopen.2023.49544)
Supplement: Supplement 2. — Data Sharing Statement [file jamanetwopen-e2349544-s002.pdf]

Data Sharing Statement

Taylor. Syndromic Antibiograms and Nursing Home Clinicians' Antibiotic Choices for Urinary Tract Infections. *JAMA Netw Open*. Published December 27, 2023.
doi:10.1001/jamanetworkopen.2023.49544

Data
Data available: Yes
Data types: Deidentified participant data
How to access data: request data access to ltaylor@medicine.wisc.edu
When available: With publication

Supporting Documents
Document types: Statistical/analytic code
How to access documents: request R code access to ltaylor@medicine.wisc.edu
When available: With publication

Additional Information
Who can access the data: anyone requesting the data
Types of analyses: re-analysis
Mechanisms of data availability: after approval of a proposal
